# Recent developments in PD-1/PD-L1 blockade research for gastroesophageal malignancies

**DOI:** 10.3389/fimmu.2022.1043517

**Published:** 2022-11-24

**Authors:** Meng Chen, Chenyan Li, Mingjun Sun, Yiling Li, Xuren Sun

**Affiliations:** ^1^ Department of Gastroenterology, First Affiliated Hospital of China Medical University, Shenyang, China; ^2^ Department of Endocrinology and Metabolism, First Affiliated Hospital of China Medical University, Shenyang, China

**Keywords:** PD-1, PD-L1, immune checkpoint inhibitors, gastric cancer, esophageal cancer, biomarkers

## Abstract

Gastroesophageal cancers (GECs) comprise malignancies in the stomach, esophagus, and gastroesophageal junction. Despite ongoing improvements in chemoradiotherapy, the clinical outcomes of GEC have not significantly improved over the years, and treatment remains challenging. Immune checkpoint inhibitors (ICIs) have been the subject of clinical trials worldwide for several years. Encouraging results have been reported in different countries, but further research is required to apply ICIs in the clinical care of patients with GEC. This review summarizes completed and ongoing clinical trials with programmed death 1 (PD-1)/programmed death-ligand 1 (PD-L1) pathway blockers in GEC and current biomarkers used for predicting PD-1/PD-L1 blockade efficacy. This review captures the main findings of PD-1/PD-L1 antibodies combined with chemotherapy as an effective first-line treatment and a monotherapy in second-line or more treatment and in maintenance therapy. This review aims to provide insight that will help guide future research and clinical trials, thereby improving the outcomes of patients with GEC.

## Introduction

Among gastroesophageal cancers (GECs), gastric cancer (GC) ranks fifth in incidence and fourth in mortality worldwide, and the median survival for advanced GC is less than 12 months (1, 2). Esophageal cancer (EC), another GEC, ranks seventh in incidence and sixth in overall mortality worldwide (1). In 2018, an estimated 570,000 individuals were diagnosed with EC worldwide, representing 3.2% of all cancer diagnoses and 5.3% of all cancer-related deaths (3). Over the past 30 years, the clinical benefits of conventional and emerging therapies have reduced GC mortality but have not improved EC survival (4, 5). In certain western nations, adenocarcinoma has overtaken squamous cell carcinoma as the most prevalent type of EC, and its incidence continues to increase in other nations (6).

During the initiation of cellular immunity, antigens presented by the major histocompatibility complex on the surface of antigen-presenting cells (APCs) can selectively bind to cell receptors of the T-lymphocyte membrane, triggering further T-cell activation, proliferation, and differentiation. Activated T cells serve a vital function in the immune system (7). Under normal physiological conditions, programmed death 1 (PD-1), a negative costimulatory immune molecule also known as an immune checkpoint, is found on the surface of T, B, and myeloid cells. PD-1 specifically connects to programmed death-ligand 1 (PD-L1) on the surface of APCs to trigger immunosuppressive signal transduction, resulting in a decrease in T-cell activity. As cancer develops, tumor cells attach to vascular endothelial or perivascular cells, fibroblasts, and lymphocytes in the surrounding tissue, constituting the tumor microenvironment (TME) in combination with the extracellular matrix (8, 9). The TME can disrupt the dynamic balance of the organism by blocking cell apoptosis and promoting angiogenesis and cell proliferation, leading to continued tumor cell development, immune escape, and distant metastasis. Tumor cells highly express PD-L1 to strengthen the PD-1/PD-L1 pathway, thereby exhausting T cells and permitting tumor cells to evade immune surveillance. Based on this principle, PD-1/PD-L1 antibodies were established to constrain the PD-1/PD-L1 signaling pathway by binding to receptors on the surface of T lymphocytes or tumor cells in the late stages of peripheral tissue regulation of T-lymphocyte function, thereby disrupting the immune response, preventing tumor cell immune escape, and ensuring a normal immune response (10). The combined positive score (CPS), the most accepted PD-L1 scoring method, refers to the count of PD-L1-positive cells (including tumor cells, lymphocytes, and macrophages) divided by the total count of live tumor cells, multiplied by 100. The National Comprehensive Care Network (NCCN) recommends PD-L1 testing (i.e., CPS) for metastatic/advanced EC and GC.

Immunotherapy for GEC includes targeted blockade against immune checkpoints such as PD-1/PD-L1, cytotoxic T lymphocyte antigen-4 (CTLA-4), T cell immunoglobulin-3 (Tim-3), lymphocyte activation gene-3 (Lag-3) and chimeric antigen receptor T-cell (CAR-T) cell therapy; and therapeutic cancer vaccines. Among immune checkpoint inhibitors (ICIs), PD-1/PD-L1 antibodies have shown well applicability in EC and GC, thus dramatically changing the treatment outlook for these patients. An increasing number of PD-1/PD-L1 blockers have been authorized for use in EC and GC treatment. Exploring the administration conditions of known PD-1/PD-L1 inhibitors and developing new antibodies are key directions of current research, as well as evaluating and predicting PD-1/PD-L1 blockade efficacy. Although considerable research through clinical trials has been conducted in EC and GC, much less is known concerning the proper indication of the medicine and the patient selection criteria in these trials, which are often among the potential limitations of the study design. The assessment of the efficacy of PD-1/PD-L1 antibodies frequently employs biomarkers that could be used to select GC and EC patients; however, much work is yet to be discovered in this area. In this review, we present an update on and evaluate the results of current clinical trials with PD-1/PD-L1 antibodies in EC and GC and briefly describe the progress in developing common predictive biomarkers. By comparing previous clinical trials, we also highlight study design limitations that warrant consideration prior to establishing future clinical trials, with the hope of assisting patients in reaching a greater survival outcome.

## Molecular and immunological basis of esophageal cancer and gastric cancer

EC does not have clear molecular typing, but one study classified EC into low- and high-risk subtypes, which might be used as independent prognostic factors (11). The Cancer Genome Atlas (TCGA) classifies four molecular subtypes of GC: Epstein–Barr virus (EBV)-positive, high microsatellite instability (MSI-H), genomically stable (GS), and chromosomal instability (CIN) (12). PD-L1 and PD-L2 expression levels are amplified in EBV-positive GC. MSI in cancer genomes is caused by DNA mismatch repair (MMR) system deficiencies. High MSI in tumors leads to the accumulation of mutational load, which affects the tumor response to anti-PD-1 antibodies (13). The United States Food and Drug Administration (FDA) has authorized pembrolizumab for the treatment of previously treated MSI-H/mismatch repair-deficient (dMMR) solid tumors, including EC and GC (14).

EC and GC are highly immunogenic, and multiple tumor neoantigens have been identified (15, 16). Owing to characteristics such as MSI and tumor mutational burden (TMB), tumor cells are highly susceptible to multiple genetic mutations, resulting in the production of specific neoantigens (17). These neoantigens can be taken up by APCs, which deliver the neoantigen to CD8+ T lymphocytes, initiating cytotoxic T lymphocytes (CTLs) and generating a key mechanism of antitumor immunity by killing tumor cells. In the TME, inflammatory factors, lymphocytes, monocytes, macrophages, and histiocytes comprise the tumor immune microenvironment. Tumor-infiltrating lymphocytes (TILs), consisting of T, B, and natural killer (NK) cells, infiltrate heavily in esophageal squamous cell carcinoma (ESCC) and gastric adenocarcinoma (18). TILs have been confirmed to be effective and independent prognostic factors during the antitumor immune response, and PD-1 expression on TILs correlates with adverse clinical outcomes in EC (19). Increased CD8+ TIL levels have been consistently detected in PD-L1-positive EC (20). Increased CD8+ TIL levels were closely associated with better survival, lower lymph node metastases, and higher PD-L1 expression levels; the combined evaluation of CD8+ TIL and PD-L1 expression has been used to predict patient responses to PD-1/PD-L1 antibody treatment in a range of malignancies (21). Large numbers of CD20+ B cells are significantly correlated with both modest lymph node involvement and lower TNM stage as independent factors for GC prognosis (22). Moreover, tumor-associated macrophages (TAMs) can release cytokines that promote cancer cell motility and invasion (23–25). Overall, high TAM density is considered to be a negative prognostic factor in GC (26). TAMs often differentiate into M1-like TAMs with pro-inflammatory and tumor-suppressive functions and M2-like TAMs with anti-inflammatory and tumor-promoting functions (27). M1-like TAMs are an independent prognostic factor in GC, and CD68+CD163-macrophages, a group of representative M1-like TAMs, can be used as predictive biomarkers to guide PD-1/PD-L1 antibody treatment in GC (28). M2-like TAMs are involved in the inhibition of antitumor immune responses by increasing PD-L1 expression in tumors (29). Patients with EC who have high levels of M2-like TAMs had shorter overall survival (OS) (30, 31). Thus, certain TAM subgroups could have prognostic value in gastric adenocarcinoma and esophageal adenocarcinoma (EAC) (32). Finally, through a variety of cytokines, cancer-associated fibroblasts (CAFs), valuable stromal cells in the TME, contribute to the growth, progression, and metastasis of EC (33, 34). CAFs upregulate PD-L1 expression, thereby promoting cancer cell proliferation in GC (35). Furthermore, a study investigating CAFs in GC reported that extracellular matrix CAFs recruited M2-like macrophages and were associated with poor prognosis (36).

## Clinical trials exploring PD-1/PD-L1 blockade in gastroesophageal cancers

PD-1/PD-L1 inhibitors have been approved for clinical use in several countries. For example, the US FDA granted pembrolizumab, nivolumab, and dostarlimab-Gxly approval for the treatment of EC and GC under certain conditions in 2022. As a first-line therapy for ESCC, camrelizumab + chemotherapy has been approved by China in 2021. However, the findings of the few clinical trials that have tested PD-1/PD-L1 antibodies as first-line monotherapies so far are not encouraging. Chemotherapy combined with PD-1/PD-L1 antibodies is currently being investigated in clinical studies as the first-line therapeutic option. This section presents the outcomes of clinical trials with PD-1/PD-L1 antibodies in EC and GC, emphasizing progress and comparing application conditions.

### PD-1/PD-L1 blockade as first-line treatment in esophageal cancer

Radical resection is the conventional first-line treatment for EC, with or without perioperative chemotherapy (37). Advanced EC is treatable with first-line chemotherapy, with an overall poor prognosis (38). Therefore, research has concentrated on the development of inhibitors for immune checkpoints. This section focuses on clinical trials exploring PD-1/PD-L1 antibodies combined with chemotherapy and introduces the application of new PD-1 antibodies as first-line treatments for EC (**Table 1**).

**Table 1:** Clinical trials of PD-1/PD-L1 blockade as first-line treatment.

Trial	Phase	Enroll	Arm	N	mOS(m)	12mOS(%)	mPFS(m)	12mFPS(%)	ORR(%)	TRAEs(%)
**First-line treatment in EC**										
KEYNOTE-590/NCT03189719	3	749	Pembrolizumab + 5-fluorouracil + cisplatin	373	12.4	NA	6.3	NA	45	98
			Placebo + 5-fluorouracil + cisplatin	376	9.8	NA	5.8	NA	29.3	97
CheckMate 648/NCT03143153	3	970	Nivolumab + cisplatin + fluorouracil	321	13.2	54	5.8	24	47	96
			nivolumab + ipilimumab	325	12.7	54	2.9	23	28	80
			cisplatin + fluorouracil	324	10.7	44	5.6	16	27	90
ESCORT-1st/NCT03691090	3	596	camrelizumab + paclitaxel + cisplatin	298	15.3	61.5	6.9	NA	72.1	99.3
			Placebo + paclitaxel + cisplatin	297	12	49.8	5.6	NA	62.1	97
JUPITER-06/NCT03829969	3	514	Toripalimab + TP	257	17	66	5.7	27.8	69.3	99.2
			Placebo + TP	257	11	43.7	5.5	6.1	52.1	99.2
ORIENT-15/NCT03748134	3	659	Sintilimab + (paclitaxel + cisplatin)/(5-fluorouracil + cisplatin)	327	16.7	64	7.2	38	66	98
			Placebo + (paclitaxel + cisplatin)/(5-fluorouracil + cisplatin)	332	12.5	52	5.7	15	45	98
NCT03603756	2	30	Camrelizumab + liposomal paclitaxel + nedaplatin + apatinib	30	19.43	NA	6.85	NA	80	100
NCT03222440	1b	20	Camrelizumab + radiotherapy	20	16.7	63.2	11.7	47.4	74	100
NCT03732508	2	23	SHR-1316 + liposomal irinotecan + 5-fluorouracil	23	11.6	NA	8.5	NA	52.2	100
**First-line treatment in GC**										
CheckMate 649/NCT02872116	3	1581	Nivolumab + XELOX/FOLFOX	789	13.8	55	7.7	33	60	NA
			XELOX/FOLFOX	792	11.6	48	6.9	23	45	NA
ATTRACTION-4/NCT02746796	3	724	Nivolumab + SOX/CAPOX	362	17.45	NA	10.45	NA	57	98
			Placebo + SOX/CAPOX	362	17.15	NA	8.34	NA	48	97
KEYNOTE-062/NCT02494583	3	763	pembrolizumab	256	10.6	46.9	2	NA	14.8	54.3
			pembrolizumab + cisplatin + fluorouracil/capecitabine	257	12.5	52.9	6.9	NA	48.6	94
			placebo + cisplatin + fluorouracil/capecitabine	250	11.1	45.6	6.4	NA	37.2	91.8
KEYNOTE-659/NCT03382600	2b	100	Pembrolizumab + SOX	54	16.9	NA	9.4	NA	72.2	100
			Pembrolizumab + SP	46	17.1	NA	8.3	NA	80.4	100
NCT03472365	2	48	camrelizumab + CAPOX, subsequentcamrelizumab + apatinib	48	14.9	68.8	6.8	NA	58.3	100

XELOX, capecitabine and oxaliplatin; FOLFOX, leucovorin, fluorouracil, and oxaliplatin; SOX, oxaliplation + S-1; CAPOX, oxaliplation +capecitabine; SP, S-1 + cisplatin; TP, paclitaxel plus cisplatin; N, number of patients; OS, overall survival; PFS, progression-free survival; ORR, object response rate; TRAEs, treatment-related adverse events; NA, not available.

KEYNOTE-590 was the first clinical trial to evaluate the combination of PD-1 inhibition with chemotherapy as a first-line treatment for EC with significant survival benefits. In March 2021, pembrolizumab plus fluoropyrimidine- and platinum-based chemotherapy was authorized by the FDA for the first-line treatment of patients with ESCC and EAC with CPS ≥10 (category 1, requires combination with cisplatin) and CPS <10 (category 2B) (39). The KEYNOTE-590 phase 3 trial enrolled 749 patients with advanced EC or Siewert type 1 gastroesophageal junction cancer (GEJC), among which 51% of the study population had CPS ≥10. The interventions included pembrolizumab or placebo plus chemotherapy (5-fluorouracil plus cisplatin). Compared to the placebo arm, the pembrolizumab arm showed a considerably enhanced survival advantage and sustained antitumor response in the total population, advanced ESCC subgroup, and CPS ≥10 subgroup. In all three populations, the pembrolizumab arm maintained an advantage in Kaplan–Meier (KM) curves for OS, and pembrolizumab + chemotherapy treatment was roughly twice as effective as placebo + chemotherapy treatment at 24-month OS. Progression-free survival (PFS), 12-month PFS, and 18-month PFS remained superior in all three populations treated with pembrolizumab plus chemotherapy. Additionally, the pembrolizumab + chemotherapy group had approximately 15% greater overall response rate (ORR), 2.3-month greater duration of response (DoR), and a nearly 3-fold increase in 24-month DoR than the placebo + chemotherapy group. No additional adverse events (AEs) were detected, indicating the safety of pembrolizumab combined with chemotherapy (40, 41).

The CheckMate-648 study evaluated PD-1 antibody combination therapy, delivering three types of drugs to patients with ESCC (n = 970): nivolumab + chemotherapy (intravenous fluorouracil), nivolumab + ipilimumab (CTLA-4 antibody), and chemotherapy alone. In the randomized population and tumor-cell PD-L1 expression of ≥1% subgroup, the nivolumab + chemotherapy group maintained higher complete response (CR) rates and longer-lasting responses at the 13-month follow-up than the other treatment groups. The median overall survival (mOS) for >12 months of the nivolumab + ipilimumab group was 2.0–6.3 months longer than that of the chemotherapy group. In patients with tumor-cell PD-L1 expression of ≥1%, the nivolumab + chemotherapy group had a substantial PFS advantage over the chemotherapy group (6.9 vs. 4.4 months). In patients with CPS ≥1 (91%), both the nivolumab + chemotherapy [hazard ratio (HR), 0.69] and nivolumab + ipilimumab (HR, 0.76) groups achieved prolonged mOS compared with that in the chemotherapy group. The survival advantage of the nivolumab-based regimen was demonstrated in subgroups with tumor-cell PD-L1 expression of ≥1% thresholds of 1%, 5%, and 10%, all with HR <1. The AEs were mainly caused by chemotherapy (nausea, loss of appetite, and stomatitis) (42). Notably, the KEYNOTE-590 and CheckMate-648 clinical trials employed similar chemotherapy drug intensities (both included fluoropyrimidine) but did not use the same evaluation criteria for PD-L1 expression and subgroup analysis.

Camrelizumab, a monoclonal antibody against PD-1, has also been researched as a first-line combination treatment in EC. Patients enrolled in the ESCORT-1st trial received camrelizumab or placebo plus chemotherapy (paclitaxel-cisplatin). The camrelizumab arm showed a longer OS tendency than the placebo arm (mOS, 15.3 vs. 12.0 months). Fewer grade 3–4 treatment-related adverse events (TRAEs) in the camrelizumab + chemotherapy group compared with the placebo + chemotherapy group (63.4% vs. 67.7%) indicated lower toxicity, with the former group experiencing adverse immune reactions mainly due to reactive capillary endothelial proliferation often associated with camrelizumab (43). The findings of this clinical trial supported the approval of camrelizumab in China for first-line treatment of unresectable, locally advanced/recurrent, or metastatic ESCC.

Toripalimab, an immunoglobulin G (IgG) PD-1 antibody, was evaluated in the JUPITER-06 trial, which enrolled 514 Chinese patients with advanced ESCC who received either toripalimab or placebo plus chemotherapy (paclitaxel plus cisplatin). PD-L1 expression was categorized as CPS ≥1 (PD-L1-positive) or CPS ≥10 (PD-L1 high expression). The toripalimab arm showed improved median progression-free survival (mPFS) (HR, 0.58) and mOS (HR, 0.58) compared to the placebo arm. The KM curves for PFS diverged early, with toripalimab retaining an advantage over the placebo. The 12-month PFS was nearly four times greater in the toripalimab + chemotherapy arm than in the placebo + chemotherapy arm. In terms of the antitumor response, the ORR (69.3% vs. 52.1%, p = 0.001) and DoR (5.6 vs. 4.2 months) were considerably higher in the toripalimab arm than in the placebo arm. The safety profile of toripalimab was considered to be acceptable. The OS and PFS benefits of toripalimab with chemotherapy were statistically significant and independent of PD-L1 expression levels (44). Both the JUPITER-06 and ESCORT-1st trials enrolled Chinese ESCC patients only. However, the survival benefit in the ESCORT-1st trial corresponded with PD-L1 expression levels, in contrast to the JUPITER-06 trial. Different PD-L1 detection methods and scoring criteria may have affected the results.

Sintilimab is a human IgG4 anti-PD-1 monoclonal antibody. In the multicenter ORIENT-15 trial, patients with ESCC received either sintilimab or placebo plus chemotherapy (93% cisplatin and paclitaxel, 7% cisplatin and 5-fluorouracil). Chinese patients made up 97% (n = 640) of the patients. The sintilimab arm had markedly better OS (16.7 vs. 12.5 months), PFS (7.2 vs. 5.7 months), and ORR (66% vs. 45%) than those in the placebo arm. The KM curves of OS remained distinct for the two groups from the beginning. The sintilimab arm outperformed the placebo arm by 13% and 23% for 1- and 2-year OS, respectively. Both tumor proportion score (TPS) and CPS for PD-L1 scoring were employed in the study. In the subgroup analysis, the survival advantage of sintilimab + chemotherapy was independent of PD-L1 expression levels (HR, 0.55 for TPS ≥10%; HR, 0.67 for TPS <10%; HR, 0.64 for CPS ≥10; HR, 0.62 for CPS <10) (45).

In the above clinical trials, PD-1 antibodies + chemotherapy were administered as a first-line combination therapy for EC. Although PD-1/PD-L1 antibody monotherapy has demonstrated good outcomes as a second- and third-line treatment, many challenges for its use as first-line treatment persist. The choice of the chemotherapeutic drug, patient distribution, inclusion criteria, and drug dose are factors that remain to be elucidated.

### PD-1/PD-L1 blockade as first-line treatment in gastric cancer

The most common first-line treatment for metastatic and incurable GC is systemic therapy, with oxaliplatin frequently favored over cisplatin due to its reduced toxicity (46). Targeted therapies have also been used as first-line treatments for patients with specific types of GC. Patients with Human epidermal growth factor receptor 2 (HER2)-overexpressed gastric adenocarcinoma are recommended to receive pembrolizumab in combination with trastuzumab and chemotherapy (fluoropyrimidine and platinum) as first-line therapy. This recommendation is according to the results of the KEYNOTE-811 clinical trial. This ongoing international phase 3 trial is evaluating HER2-positive GC/GEJC in 692 patients treated with pembrolizumab or placebo plus trastuzumab and chemotherapy (capecitabine + oxaliplatin or fluorouracil + cisplatin). The trial employs MSI-H and PD-L1 as biomarkers. In the study population, 84.1% of patients had CPS ≥1, and large differences in ORR were reported. In the first interim analysis of 260 patients after an 8.5-month follow-up, the pembrolizumab arm had approximately 20% greater ORR than the placebo arm (74.4% vs. 51.9%) and maintained certain advantages in CR, disease control rate (DCR), and DoR, suggesting a more robust and durable response. Among the 433 patients examined for safety, the pembrolizumab group showed a lower incidence of grade 3–5 AEs and AEs leading to death than the placebo group. We look forward to updates from this trial (47, 48).

Based on the excellent clinical benefits and durable response achieved by nivolumab in combination with fluoropyrimidine- and platinum-containing chemotherapy in patients suffering from unresectable HER2-negative GC, GEJC, and EAC, the FDA approved this therapy in April 2021 for first-line treatment of tumors with CPS ≥5 (category 1) and CPS <5 under certain circumstances (category 2B) (49). In the CheckMate-649 trial, the analysis of survival status and antitumor response was divided into CPS ≥1 and CPS ≥5 subgroups. The nivolumab arm achieved a more pronounced OS benefit than the chemotherapy arm in the CPS ≥5 cohort (mOS, 14.4 vs. 11.1 months), CPS ≥1 cohort (HR, 0.77), and in all random patients (HR, 0.80). In patients with CPS ≥5, the nivolumab arm had 1.7-month longer PFS than the chemotherapy arm (7.7 vs. 6.0 months) and 14% longer 1-year PFS. The follow-up study determined that the survival benefit of nivolumab + chemotherapy increased with higher CPS cutoff value. In patients with CPS ≥5, the nivolumab + chemotherapy group had 15% greater ORR and 2.5-month longer response duration than the chemotherapy group. The advantage of an intense and prolonged response was also reflected in the randomized population. Meanwhile, as per the number needed to treat (NNT) analysis, the nivolumab + chemotherapy group maintained a consistent advantage over the chemotherapy group on the basis of OS, PFS, and ORR in the whole population and the CPS ≥5 subgroup. The prevalence of TRAEs was considerably higher in the nivolumab + chemotherapy group than in the chemotherapy alone group (22% vs. 12%) with more grade 3**–**4 TRAEs (59% vs. 44%). However, the nivolumab arm showed a lower risk of deteriorating symptoms than the chemotherapy arm (CPS ≥5, HR, 0.64; overall patients, HR, 0.77). Additionally, the nivolumab + chemotherapy group was associated with improved quality-adjusted time without symptoms or toxicity (Q-TWiST) compared to the chemotherapy group. Improving quality of life (QOL) also helps clinicians better manage patients (50–52).

A similar trial, ATTRACTION-4, enrolled 724 Asian patients with GC/GEJC from Japan, Korea, and Taiwan. The trial evaluated either nivolumab or placebo plus chemotherapy (oxaliplatin + capecitabine or fluoropyrimidine S-1). Although the OS between the two arms did not differ significantly (p = 0.26), the mPFS of the nivolumab arm was nearly 2 months longer than that of the placebo arm (10.45 vs. 8.34 months; HR, 0.68). The KM curves for PFS separated early, and the nivolumab arm consistently had superior PFS rates than the placebo arm. Additionally, regardless of PD-L1 expression levels, the nivolumab arm had a better antitumor response. The ORR was nearly 10% greater in the nivolumab arm than that in the placebo arm (57% vs. 48%). The nivolumab arm was associated with improved survival and 4-month longer DoR than the placebo arm (12.91 vs. 8.67 months). Although the nivolumab + chemotherapy group had more frequent TRAEs than the placebo + chemotherapy group, including grade ≥3 TRAEs, serious TRAEs, and TRAEs leading to treatment discontinuation, the types of TRAEs were consistent with those previously associated with chemotherapy and nivolumab treatment. The researchers determined that the toxicity of chemotherapy plus nivolumab was manageable, and that nivolumab combined with chemotherapy helped maintain QOL (53, 54). Compared to the CheckMate 649 trial, the ATTRACTION-4 trial enrolled Asian patients only and had more patients receiving subsequent anticancer drugs, which may be one of the reasons for the mOS difference between trials. Both trials added oxaliplatin as a chemotherapeutic agent and achieved good results, indicating that oxaliplatin works well in combination with nivolumab.

Pembrolizumab monotherapy was also explored as a first-line treatment for GC. The KEYNOTE-062 trial was established based on the positive outcomes of the KEYNOTE-059 and KEYNOTE-060 trials; however, KEYNOTE-062 did not achieve the desired results. The GC/GEJC population with CPS ≥1 was allocated to three arms: pembrolizumab or placebo plus chemotherapy (cisplatin combined with fluorouracil/capecitabine) and pembrolizumab alone. Analyses were performed based on CPS ≥10 (n = 281) and MSI-H (n = 50) subgroups. Among the overall study population with CPS ≥1, the pembrolizumab arm showed a lower OS compared with the chemotherapy arm (HR, 0.91) but approximately 1% and 6% higher 1- and 2-year OS, respectively. Pembrolizumab had a survival advantage over chemotherapy (HR, 0.91) and induced a longer DoR (13.7 vs. 6.8 months), suggesting that pembrolizumab had a long-term beneficial effect. In the CPS ≥10 cohort (n = 281), the pembrolizumab monotherapy arm seemed to have a clinical advantage over the chemotherapy arm, although the difference was not tested statistically (mOS, 17.4 vs. 10.8 months; HR, 0.62). The pembrolizumab arm had fewer TRAEs (54.3% vs. 91.8%) and grade ≥3 TRAEs (16.9% vs. 69.3%) than the chemotherapy arm. The overall population with CPS ≥1 was able to maintain health-related quality of life (HRQOL) when treated with pembrolizumab alone or pembrolizumab plus chemotherapy. A correlation between clinical efficacy and TMB in the pembrolizumab arm was proposed at a later stage of the study. The findings remained consistent at the 54.3-month follow-up, with the CPS ≥1 and CPS ≥10 subgroups treated with pembrolizumab having 8% and 18% greater 2-year OS than those treated with chemotherapy, respectively (55–58). Despite the lack of survival benefits compared to chemotherapy, pembrolizumab achieved better clinical benefit in the CPS ≥10 cohort than in the CPS ≥1 subgroup, suggesting that increased PD-L1 expression levels may improve OS for patients with GC. These findings seemed comparable to those in the CheckMate 649 trial. In contrast to the KEYNOTE-811 and ATTRACTION-4 trials, the KEYNOTE-062 trial used cisplatin rather than oxaliplatin, which may have led to differences in outcomes. In the ongoing KEYNOTE-859 trial, researchers are exploring the clinical effectiveness of pembrolizumab in combination with chemotherapy using 5-fluorouracil + cisplatin or capecitabine + oxaliplatin as the chemotherapeutic agents (59).

More trials investigating the combination of PD-1/PD-L1 antibodies and chemotherapy for GC/GEJC treatment are ongoing. The ORIENT-16 trial is exploring the clinical efficacy of sintilimab + oxaliplatin + capecitabine (60). The BGBA317305 trial (NCT03777657) is investigating the clinical efficacy of tislelizumab in combination with oxaliplatin + capecitabine or cisplatin + 5 fluorouracil (61). The above clinical trial results highlight that chemotherapy remains the mainstream first-line combination treatment for EC and GC for the time being. Studies exploring PD-1 antibody monotherapies have not yet demonstrated clinical advantages; however, the impact of different PD-L1 expression cutoffs on patient outcomes may influence future ICI studies.

### PD-1/PD-L1 blockade as second-line or more treatment in esophageal cancer

Abundant PD-1/PD-L1 antibodies are involved in second-line treatment studies of EC and GC. Both monotherapies and combination therapies have demonstrated good applicability, and research is now focused on the possible applications of PD-1 antibody monotherapy as second-line or more treatments. Many of these agents have been approved by the FDA, including pembrolizumab, which has been approved for previously treated unresectable/metastatic MSI-H/dMMR or TMB-H solid tumors, including EC and GC (62, 63). Dostarlimab-Gxly is a second-line or more therapeutic option for MSI-H/dMMR GEC (64). Meanwhile, nivolumab is recommended for advanced ESCC (category 1), and pembrolizumab is also recommended for advanced ESCC with CPS ≥10 (category 1) (**Table 2**).

**Table 2:** Clinical trials of PD-1/PD-L1 blockade in first-maintenance or second-line treatment.

trail	phase	enroll	arm	N	mOS(m)	12mOS(%)	mPFS(m)	12mFPS(%)	ORR(%)	TRAEs(%)
**second-line treatment or more in EC**										
KEYNOTE-180/NCT02559687	2	121	Pembrolizumab	121	5.8	28	2	NA	9.9	57.9
										
KEYNOTE-181/NCT02564263	3	628	pembrolizumab	314	7.1	32.4	2.1	NA	13.1	64
			paclitaxel/docetaxel/irinotecan	297	7.1	24.2	3.4	NA	6.7	86
										
RATIONALE-302/NCT03430843	3	512	tislelizumab	256	8.6	37.4	1.6	12.7	20.3	73.3
			paclitaxel/docetaxel/irinotecan	256	6.3	23.7	2.1	1.9	9.8	93.8
										
ORIENT-2/NCT03116152	2	190	sintilimab	95	7.2	37.4	1.6	10.4	12.6	54.3
			paclitaxel/irinotecan	95	6.2	21.4	2.9	1.7	6.3	90.8
										
ESCORT/NCT03099382	3	457	camrelizumab	228	8.3	34	1.9	10	NA	94
			docetaxel/irinotecan	220	6.2	22	1.9	NA	NA	90
										
ATTRACTION-3/NCT02569242	3	419	nivolumab	210	10.9	47	1.7	12	NA	65
			paclitaxel/docetaxel	209	8.4	34	3.4	7	NA	95
										
ATTRACTION-1/ONO-4538-;07	2	65	nivolumab	64	10.8	45.2	1.5	10.3	17.2	63.1
										
NCT02971956	2	49	Pembrolizumab	49	5.8	31.9	1.84	4.1	8	78
										
**first-line maintenance treatment in GC**										
JAVELIN Gastric 100/NCT02625610	3	499	avelumab	249	10.4	NA	3.2	NA	13.3	61.3
			continued chemotherapy	250	10.9	NA	4.4	NA	14.4	77.3
							
										
JAVELIN Solid Tumor trial/NCT01772004	1b	150	1 L-mn avelumab	90	11.1	46.2	2.8	13	6.7	63.3
			1 L chemotherapy		18.7	31.7	NA	NA	6.7	
			2 L avelumab	60	6.6	25.6	1.4	2	6.7	46.7
										
**second-line treatment or more in GC**										
KEYNOTE-059/NCT02335411	2	259	pembrolizumab	259	5.6	23.4	2	NA	11.6	60.2

											
KEYNOTE-061/NCT02370498	3	592	pembrolizumab	296	9.1	40	1.5	14	NA	53	
			paclitaxel	296	8.3	27	4.1	9	NA	84	
											
KEYNOTE-063 /NCT03019588	3	94	pembrolizumab	47	8	NA	2	NA	13	60	
			paclitaxel	47	8	NA	4	NA	19	96	
											
ATTRACTION-2/ONO-4538-12/NCT02267343	3	493	nivolumab	330	5.26	26.2	1.61	7.6	11.2	43	
			placebo	163	4.14	10.9	1.45	1.5	0	27	
											
JAVELIN Gastric 300/NCT02625623	3	371	avelumab	185	4.6	NA	1.4	NA	2.2	48.9	
			chemotherapy	186	5	NA	2.7	NA	4.3	74	
											
CheckMate-032/NCT01928394	1/2	160	Nivolumab 3mg/kg	59	6.2	39	1.4	8	12	69	
			Nivolumab 1mg/kg plus ipilimumab 3mg/kg	49	6.9	35	1.4	17	24	84	
			Nivolumab 3mg/kg plus ipilimumab 1mg/kg	52	4.8	24	1.6	10	8	75	

1 L, First-Line; 1L-mn, First-Line Maintenance; 2 L, Second-Line; N, Number of patients; OS, Overall Survival; PFS, Progression Free Survival; ORR, Object Response Rate; TRAEs, Treatment-Related Adverse Events; NA, Not Available.

Based on the positive outcomes of the KEYNOTE-180 and KEYNOTE-181 trials, the FDA approved pembrolizumab in 2019 as a second-line treatment for locally advanced/metastatic ESCC with CPS ≥10 (65). The phase II KEYNOTE-180 trial enrolled patients with advanced ESCC (n = 63) or EAC who had undergone second-line or more treatment, and patients were administered pembrolizumab for subsequent treatment. PD-L1-positive expression was defined as CPS ≥10. Antitumor responses were observed in the overall population (ORR, 9.9%), CPS ≥10 subgroup (ORR, 13.8%), and CPS <10 subgroup (ORR, 6.3%). Pembrolizumab conferred a significant survival advantage (OS, 5.8 months; 6-month OS, 49%; 12-month OS, 28%) and was deemed to be safe (TRAEs, 12.4%). The results suggested that PD-L1 expression levels may enhance the response to pembrolizumab in patients with ESCC or EAC (66, 67). In the subsequent multicenter KEYNOTE-181 trial, 528 patients (63.9%) were treated with pembrolizumab or chemotherapy (irinotecan, paclitaxel, or docetaxel). The survival advantage of pembrolizumab was more pronounced than that of chemotherapy for Asian patients. Additionally, pembrolizumab did not prolong mOS in all patients but presented a notable survival benefit in the CPS ≥10 subgroup. Among the CPS ≥10 cohort, the pembrolizumab arm had an OS advantage of almost 2.6 months over the chemotherapy arm (9.3 vs. 6.7 months), 20% greater 1-year OS (43.0% vs. 20.4%), and reduced risk of death (PFS, HR, 0.73). Among patients with ESCC, the 12-month PFS increased by 7% (16.7% vs. 7.4%). The most significant improvement in survival was observed in patients with ESCC with CPS ≥10 (HR, 0.64). An antitumor response advantage was reported in the pembrolizumab arm over the chemotherapy arm in the patients with ESCC (ORR, 16.7% vs. 7.4%), CPS ≥10 subgroup (ORR, 21.5% vs. 6.1%), and the randomized population (ORR, 13.1% vs. 6.9%). The 9-month response rate to pembrolizumab was higher than that to chemotherapy (53.5% vs. 38.1%), indicating a longer duration of response. The pembrolizumab arm had almost 20% fewer TRAEs and grade ≥3 TRAEs than the chemotherapy arm, and both sets of patients had similar HRQOL values, suggesting that pembrolizumab had a superior safety profile. However, the cost of pembrolizumab treatment far exceeded that of chemotherapy by $37,201.68. Health practitioners may value the application of pembrolizumab as a second-line therapy for EC (68–70). Both trials supported pembrolizumab monotherapy as a second-line treatment for EC. Furthermore, pembrolizumab showed greater efficacy in ESCC.

A growing number of newly developed PD-1 antibody single agents are being investigated in ESCC, and most trials have been conducted in China, where ESCC is the major subtype of EC. In the multicenter RATIONALE-302 trial, tislelizumab or chemotherapy (irinotecan, docetaxel, or paclitaxel) were administered to patients with metastatic or advanced ESCC. Tislelizumab is a specific antibody designed to target PD-1. PD-L1 expression was estimated using tumor area positivity (TAP), with TAP ≥10% set as the criterion for positive PD-L1 expression. In the overall population, the tislelizumab arm displayed an OS advantage over the chemotherapy arm (8.6 vs. 6.3 months; HR, 0.70). The mPFS was shorter in the tislelizumab arm than in the chemotherapy arm, but the KM curves for PFS began to separate at 3 months and the PFS rates for the tislelizumab arm remained progressively higher than those of the chemotherapy arm (6-month PFS, 21.9% vs. 14.9%; 12-month PFS, 12.7% vs. 1.9%). The tislelizumab arm had an OS advantage over the chemotherapy arm in the TAP ≥10% subgroup (10.3 vs. 6.8 months; HR, 0.54), TAP <10% subgroup (HR, 0.82) and TAP unknown subgroup (HR, 0.67). The OS advantage was demonstrated regardless of PD-L1 expression levels, as determined by *post-hoc* interaction analysis. The ORR of the tislelizumab arm was 10% higher than that of the chemotherapy arm (20.3% vs. 9.8%), indicating a longer-lasting antitumor response. The tislelizumab arm experienced fewer TRAEs and grade ≥ 3 TRAEs than the chemotherapy arm. Patients with advanced ESCC treated with tislelizumab demonstrated clinical improvement in OS (HR, 0.70) and a lower decline in physical function, leading to extended HRQOL (71, 72).

The phase 2 ORIENT-2 trial explored sintilimab as a second-line monotherapy for ESCC. The trial enrolled 190 patients with metastatic or advanced ESCC who were randomly assigned to the sintilimab or chemotherapy (paclitaxel or irinotecan) arms of the study. The mOS of the sintilimab arm was 1 month longer than that of the chemotherapy arm (7.2 vs. 6.2 months; HR, 0.70). The survival advantage of sintilimab over chemotherapy showed a longer tendency in the 12-month OS (37.4% vs. 21.4%) and 12-month PFS (10.7% vs. 1.9%). The sintilimab arm also had a superior safety profile than the chemotherapy arm (grade ≥3 TRAEs, 20.2% vs. 39.1%). The restricted mean survival time (RMST) and Fleming–Harrington tests led to the conclusion that sintilimab treatment for ESCC was associated with prolonged response and possible long-term survival. Biomarker analysis revealed that patients with a low neutrophil-to-lymphocyte ratio (NLR) (NLR <3) 6 weeks after sintilimab treatment had a substantial survival benefit over those with NLR >3 (OS, 14.0 vs. 6.2 months; PFS, 2.9 vs. 1.5 months). Moreover, low molecular tumor burden index (mTBI) in peripheral blood was associated with PFS (HR, 0.55), demonstrating the clinical significance of mTBI in sintilimab-treated patients. Based on these findings, researchers recommended the combination of low mTBI with high T-cell receptor clonality and NLR <3 at 6 weeks after treatment as biomarkers for predicting survival outcomes (OS and PFS) of sintilimab-treated patients with ESCC (73).

In addition to these trials, the ESCORT trial investigated camrelizumab monotherapy as a second-line treatment for advanced/metastatic ESCC in China (74), while the ATTRACTION-3 trial explored nivolumab monotherapy as a second-line therapy for advanced/metastatic ESCC (75). The above trials supported the popularity of PD-1 antibodies as monotherapies in second-line or more therapy studies in EC because Asian patients accounted for the majority of participants in these studies. In addition, regional differences were reflected in the KEYNOTE-181 study with Asian patients benefiting more from PD-1 blockade treatment than non-Asian patients, although the RATIONALE-302 trial did not report the same results. Additionally, different trials used different PD-L1 expression criteria, and the ORIENT-2 trial did not predict the absolute benefit of sintilimab treatment despite the use of both TPS and CPS. The exploration of appropriate predictive markers remains a pending issue.

### PD-1/PD-L1 blockade as first-line maintenance therapy and second-line or more treatment in gastric cancer

Unlike EC, nivolumab and pembrolizumab monotherapies have not been authorized by the FDA as second-line treatments for GC. The conventional second-line treatment for GC is ramucirumab alone or in combination with paclitaxel (76); single-agent paclitaxel, docetaxel, and irinotecan are also suggested as category 1 therapies.

The phase 3 JAVELIN Gastric 100 trial explored the clinical effectiveness of avelumab applied to GC/GEJC as a maintenance therapy after primary induction chemotherapy. Avelumab did not markedly improve OS in either the PD-L1 expression on ≥1% of tumor cells (defined as PD-L1-positive) subgroup or randomized population. The KM curves for OS were lower in the avelumab arm than in the chemotherapy arm until 12 months. However, once the two curves crossed over, the avelumab arm preserved a trend toward higher OS, outperforming the chemotherapy arm by approximately 6% at 24-month OS (22.1% vs. 15.4%). The 1-year DoR and 2-year responses for the avelumab arm were approximately two and four times longer than those for the chemotherapy arm, respectively. In the CPS ≥1 subgroup, the mOS was comparatively higher in the avelumab arm than in the chemotherapy arm (HR, 0.72). Grade ≥3 AEs, TRAEs, and severe TRAEs occurred less frequently in the avelumab arm than in the chemotherapy arm. Although the JAVELIN Gastric 100 trial did not reach the primary endpoint of OS improvement, the potential survival benefits and excellent safety profile of avelumab in long-term treatment are informative (77). The JAVELIN Solid Tumor trial (78) also investigated the efficacy of avelumab as a first-line maintenance therapy for tumors. Although the trial data did not show a significant advantage over chemotherapy, the favorable 12-month OS and PFS in the JAVELIN Solid Tumor trial suggest a lasting effect of avelumab in long-term first-line maintenance treatment for patients with GC.

As a second-line treatment, pembrolizumab monotherapy in the phase 2 KEYNOTE-059 trial demonstrated good efficacy in advanced GC/GEJC. The phase 3 KEYNOTE-061 trial enrolled 395 patients with GC/GEJC with CPS ≥1 for subsequent administration of pembrolizumab or chemotherapy (paclitaxel). In the overall population, pembrolizumab did not demonstrate superiority in terms of OS (HR, 0.82). In the long-term follow-up, the KM curves separated at 8 months, after which the pembrolizumab arm had greater 12-month (13%) and 18-month (11%) OS than that in the chemotherapy arm. The superior response time of the pembrolizumab arm compared to the chemotherapy arm (18.0 vs. 5.3 months) suggests a survival advantage in long-term therapy. In the CPS ≥10 cohort, the OS of the pembrolizumab arm was 2.4 months longer than that of the chemotherapy arm (HR, 0.64). Pembrolizumab was associated with fewer toxic events than paclitaxel, including TRAEs, grade ≥3 TRAEs, and AEs leading to treatment discontinuation. The pembrolizumab and paclitaxel arms had comparable HRQOL scores. In the CPS ≥1 subgroup, the pembrolizumab arm had prolonged mOS compared to the paclitaxel arm (HR, 0.81), and the pembrolizumab arm had approximately 15% greater ORR than the paclitaxel arm in the CPS ≥10 cohort. The difference in 2-year OS between the pembrolizumab and paclitaxel arms increased with increasing CPS cutoff values (CPS ≥5, 15.4%; CPS ≥10, 21.1%). Additionally, the efficacy of pembrolizumab (PFS and ORR) progressively improved with increasing PD-L1 expression levels. In the CPS ≥1 subgroup, patients with Eastern Cooperative Oncology Group performance status (ECOG PS) 0 fared better when treated with pembrolizumab than with paclitaxel (OS, 12.3 vs. 9.3 months), with different results observed for patients with ECOG PS 1 (OS, 5.4 vs. 7.5 months). These results suggest that patients with better ECOG PS may respond more favorably to pembrolizumab treatment. In the follow-up biomarker analysis, tissue TMB was suggested as a predictor of pembrolizumab treatment in GC, but there are also conflicting views (79–85). Both the KEYNOTE-061 and KEYNOTE-062 trials achieved good and durable survival benefits in the CPS ≥10 subgroup, suggesting that patients with GC with high levels of PD-L1 expression may better respond to pembrolizumab, further supporting the use of PD-1 antibodies for patients with GC. The newly launched phase 3 KEYNOTE-063 trial was conducted after the KEYNOTE-061 trial. The KEYNOTE-063 trial enrolled 94 patients with advanced GC/GEJC with CPS ≥1 in Asia. This trial revealed superior results for the safety of pembrolizumab, although no definitive conclusions were reached regarding survival status and antitumor response (86).

The use of PD-1 antibodies as second-line or more treatments in GC is worth further exploration. Both the ATTRACTION-2 and CheckMate-032 trials included nivolumab, and the results were of relative clinical value, while nivolumab in the CheckMate-032 had better clinical value than nivolumab plus ipilimumab, suggesting that nivolumab-related studies are deserving of future exploration. Nevertheless, further consideration needs to be given to appropriate control treatments, since conventional second-line chemotherapy drugs may be more comparable than placebo treatments.

### PD-1/PD-L1 blockade as perioperative treatment

Combined treatment improves patient survival more than resection alone in patients with localized EC or esophagogastric junction cancer (EGJC) (87, 88). Both perioperative and preoperative chemotherapy are routine regimens (89, 90). Based on the findings of the CheckMate 577 trial, nivolumab monotherapy was licensed by the FDA in May 2021 for patients with residual disease following preoperative chemoradiation and R0 resection (category 1) (91). In the CheckMate 577 trial, patients with EC/GEJC who received neoadjuvant radiotherapy were recruited and given either nivolumab or switched to a placebo treatment schedule. PFS was roughly twice as long in the nivolumab arm as that in the placebo arm (22.4 vs. 11.0 months; HR for disease recurrence or death, 0.69). The two arms continued to diverge in the KM curves, with nivolumab being continuously superior to the placebo. More AEs were associated with nivolumab treatment than with placebo treatment, but the safety profile was consistent with that of earlier trials. In the subgroup analysis, similar HR values for disease recurrence or mortality were observed for tumor-cell PD-L1 expression ≥1% (HR 0.75) and <1% (HR, 0.73), indicating that the efficacy of adjuvant nivolumab treatment was independent of PD-L1 expression levels (92). According to the CheckMate 577 trial, the European Society of Molecular Oncology recommends nivolumab as standard therapy for patients with EC/GEJC undergoing neoadjuvant chemoradiotherapy, regardless of histologic subtype (93).

Localized GC can also be treated with combination therapy to improve survival. Clinical trials exploring PD-1 antibodies combined with chemotherapy as a neoadjuvant therapy in GC have been conducted. A phase 2 study explored neoadjuvant treatment with capecitabine, sintilimab, and oxaliplatin in locally advanced GC/GEJC before surgical resection. A pathological complete response (pCR) was considered to be a predictor of the long-term benefit of neoadjuvant treatment and was set as the primary endpoint of the study. pCR and major pathological response (MPR) was achieved in 19.4% and 47.2% of the study population, respectively. The researchers attributed the results to the multiple drug combination and a high proportion of the study population with CPS ≥1. The CPS ≥1 subgroup had higher pCR (28.6%) and MPR (57.1%) than the overall population, supporting the use of CPS as a predictive biomarker to screen those who might best benefit from neoadjuvant anti-PD-1 therapy (94). Although not as much attention has been given to PD-1 antibodies in neoadjuvant studies as in first- and second-line treatment studies, many trials are underway. For instance, the KEYNOTE-585 trial has confirmed the effectiveness of perioperative chemotherapy in combination with pembrolizumab in GC (95).

## Predictive biomarkers of PD-1/PD-L1 blockade efficacy

As seen from the above clinical trials, many conditions limit the ability of PD-1/PD-L1 blockade to achieve good results, and a considerable number of patients do not respond to therapy. Predictive biomarkers are essential for screening patients before the start of treatment and avoiding adverse effects. This section presents a short summary of common biomarkers used in clinical trials and briefly introduces those that may predict the effectiveness of PD-1/PD-L1 antibodies.

PD-L1 and MSI-H are recommended by the NCCN as common biomarkers in GC and EC. As shown in multiple clinical trials, patients with different PD-L1 expression levels often exhibit differences in response to PD-1 antibodies. In the CheckMate 032 trial, the beneficial effects of nivolumab in combination with ipilimumab increased with higher CPS levels, suggesting the superiority of CPS as a biomarker (96). Although the effectiveness of PD-1 antibodies in some trials was independent of PD-L1 expression levels, this difference may stem from different PD-L1 detection methods, evaluation criteria, and location of the patient. As common molecular subtypes, EBV-positive GC and MSI-H GC were both associated with enhanced ORR and PD-L1/PD-1 antibody efficacy, with EBV-positive GC having close to 100% ORR (28). Patients with MSI-H GC may have shorter PFS and lower ORR when receiving first-line chemotherapy, but higher ORR and PFS was achieved after subsequent PD-1 antibody treatment, supporting the early use of ICIs in MSI-H GC (97). Genome sequencing demonstrated that both EBV-positive GC and MSI-H GC were associated with high PD-L1 expression levels and favorable response to pembrolizumab (98).

Other common biomarkers have also been explored in GC and EC. TMB is associated with better response to PD-1 antibody treatment in EC (99). NLR is one of the leading predictive indicators of nivolumab efficacy in GC, providing a straightforward, easily acquired, and cost-effective biomarker (100). Changes in the gut microbiome were found in the DELIVER trial, in which the mechanism for bacterial invasion of epithelial cells was related to nivolumab clinical outcomes and progressive disease, suggesting a potential novel biomarker for predicting treatment response to nivolumab in advanced GC (101). Numerous predictive biomarkers have been investigated in clinical trials of GC and EC, but practical biomarkers need to be validated by credible findings.

## Conclusions and perspectives

The standard of care for EC and GC has long revolved around chemotherapy and surgery. Along with research progress in targeted therapies, PD-1/PD-L1 antibodies continue to be investigated in clinical trials as reliable ICIs. This review presents an overview of the molecular and immunological background of PD-1/PD-L1 antibody applications, summarizes recent clinical trials investigating PD-1/PD-L1 blockade in EC and GC/GEJC, and briefly introduces common predictive biomarkers that could be further investigated. However, the clinical trials described herein have various potential problems that complicate the evaluation of their results. For example, some trials specified PD-L1 expression levels as an inclusion criterion, whereas other trials only explored PD-L1 expression in subgroup analyses. Furthermore, subgroups with different CPS cutoff values yielded varied CPS scores for survival results, while different PD-L1 expression detection methods might further skew conclusions when comparing trial results. Moreover, small disparities between patient locations, cancer types, and control groups affected trial outcomes and the ability to draw meaningful conclusions across trials. Indeed, the proportion of Asian patients in the study population may affect study outcomes. In addition, some chemotherapeutic drugs may affect the TME and impact the effectiveness of PD-1/PD-L1 antibodies (102, 103). Although PD-1/PD-L1 antibody treatment can prolong the life of some patients with GEC, the increased incidence of adverse effects when combined with chemotherapy cannot be ignored, and patients may develop a reduced tolerance to the drug, thereby risking treatment discontinuation. Finally, PD-1/PD-L1 antibodies are more expensive than conventional treatments, and both PD-L1 testing and dosing portals increase the cost of patient treatment. The above issues should be considered by investigators when designing future trials.

As immunotherapy research continues to advance, we believe that modalities of PD-1/PD-L1 blockade in EC and GC will further evolve. Here, we review and advise on common related issues (**Table 3**). First-line treatment in EC and GC has been extensively studied in combination with chemotherapy, and the choice of chemotherapeutic agents has been compared for effectiveness, while treatment alone has not yielded good results. Along with radiotherapy (104), CTLA-4 (ipilimumab), HER2 [trastuzumab (105) and margetuximab], and vascular endothelial growth factor receptor-2 (VEGFR-2) (106) antibodies are also being explored in clinical trials; studies on PD-1/PD-L1 in combination with other therapeutic modalities are promising. In response to the poor results of classical PD-1 antibody in a first-line trial, it is possible to investigate the application of PD-1 monotherapy in a strictly screened range of patients, such as PD-L1 CPS cutoffs, molecular subtypes, pathological types, and immune cell levels. Moreover, studies of biomarker detection can be performed in parallel with trials on subgroup analysis. Many PD-1 antibodies have been used in clinical studies for second-line therapy, but only pembrolizumab is used as the first choice in CPS ≥10 ESCC, with the others suggested as second-line treatment options. Other PD-1 antibodies might be tested in trials to determine their suitability in a range of patients through subgroup analysis. The new PD-1 antibody tislelizumab/sintilimab monotherapy study focused mainly on Asian ESCC patients, and the new drug could be considered for validation in a large clinical trial, including EC patients worldwide. Non-Asian regions have different pathology type proportions. How to control the balance of patient proportions needs to be considered when enrolling patients in future studies. Considering that avelumab has not achieved a clear advantage in first-line maintenance therapy, conventional PD-1 antibodies could be taken into consideration. Perioperative therapy emphasizes the importance of PD-1/PD-L1 antibodies in neoadjuvant therapy, while PD-1 antibodies in neoadjuvant therapy are typically administered as a combination or monotherapy following chemotherapy. Future studies must focus on the effect of PD-1 antibodies alone and apply PD-1 antibodies to other stages of perioperative therapy. As PD-1/PD-L1 antibodies in the CPS ≥1 subgroup are analyzed effectively in neoadjuvant therapy, whether PD-L1 routine testing is applicable to patients who could receive neoadjuvant therapy should be further investigated. In terms of biomarkers, HER2, MSI-H, and PD-L1 are currently used in testing, but new potential biomarkers are needed for HER2-, MSI-H-, and PD-L1-negative patients. Bioinformatics analysis to screen tumor cell gene expression characteristics or molecular pathways, as well as cellular and cytokine changes in the TME, may provide suitable combinatorial biomarkers. Overcoming the abovementioned drawbacks and exploring the best therapeutic outcomes in patients with complex EC and GC will help future investigators design valuable clinical trials, yielding beneficial outcomes.

**Table 3:** Overview of clinical trials through comparison.

Source	Cancer types	PD-L1 scoring method and setting cut-offs	PD-1/PD-L1 antibody combined-agent or monotherapy	Results
KEYNOTE-590	EC/Siewert type 1 GEJC	CPS of 10	combined with 5-fluorouracil + cisplatin	better mOS and mPFS in patients with ESCC, patients with CPS of 10 or more and all patients
CheckMate-648	ESCC	CPS of 1 and tumor-cell PD-L1 expression of 1%	combined with cisplatin + fluorouracil	better mOS in patients with ESCC
ESCORT-1st	ESCC, all patients were Chinese	TPS of 1,5,10%	combined with paclitaxel + cisplatin	better mOS and mPFS in patients with ESCC
JUPITER-06	ESCC, all patients were Chinese	CPS of 1,10	combined with TP	better mOS and PFS benefits in patients with ESCC independent of PD-L1 expression levels
ORIENT-15	ESCC, 97% of patients was Chinese	TPS of 1,5,10% and CPS 1,5,10	combined with (paclitaxel + cisplatin)/(5-fluorouracil + cisplatin)	better mOS and PFS benefits in patients with ESCC independent of PD-L1 expression levels
KEYNOTE-811	HER2-overpressed GC/GEC	CPS of 1, 84.1% of patients had CPS of 1 or more	combined with trastuzumab + (5-fluorouracil and cisplatin)/(capecitabine and oxaliplatin)	ongoing
CheckMate 649	HER2-negative GC/GEJC/EAC	CPS of 1,5	combined with XELOX/FOLFOX	better mOS and mPFS in patients with CPS of 5 or more and all patients
ATTRACTION-4	GC/GEJC, all patients were Asian	tumor-cell PD-L1 expression of 1%	combined with SOX/CAPOX	better mPFS in all patients
KEYNOTE-062	GC/GEJC with CPS of 1 or more	CPS of 1,10	combined with cisplatin + fluorouracil/capecitabine	not-positive results
KEYNOTE-180	EC	CPS of 10	monotherapy	PD-L1 expression levels may enhance the response to pembrolizumab in patients with ESCC or EAC
KEYNOTE-181	EC	CPS of 10	monotherapy	better mOS in patients with ESCC and patients with CPS of 10 or more
RATIONALE-302	ESCC	TAP of 10%	monotherapy	better mOS in all patients independent of PD-L1 expression levels
ORIENT-2	ESCC, all patients were Chinese	TPS of 1,10% and CPS 1,10	monotherapy	better mOS in all patients
JAVELIN Gastric 100	GC/GEJC	tumor-cell PD-L1 expression of 1%	monotherapy	not-positive results
KEYNOTE-061	GC/GEJC with CPS of 1 or more	CPS of 1	monotherapy	not-positive results, but high levels of PD-L1 expression may better respond to pembrolizumab
CheckMate 577	EC/GEJC	tumor-cell PD-L1 expression of 1%	monotherapy	better disease-free survival in all patients

ESCC, esophageal squamous cell carcinoma; GEJC, gastroesophageal junction cancer; GEC, gastroesophageal cancer; GC, gastric cancer; EC, esophageal cancer; CPS, combined positive score; TPS, tumor proportion score; TAP, tumor area positivity; XELOX, capecitabine and oxaliplatin; FOLFOX, leucovorin, fluorouracil, and oxaliplatin; SOX, oxaliplatin + S-1; CAPOX, oxaliplatin +capecitabine; SP, S-1 + cisplatin; TP, paclitaxel plus cisplatin.

## Author contributions

MC: Conceptualization, Methodology, Investigation, Writing – Original Draft. CL: Supervision. MS: Supervision. YL: Supervision. XS: Supervision, Writing – Review and Editing, Project administration. All authors contributed to the article and approved the submitted version.

## Acknowledgments

The authors would like to thank XS and CL for their valuable help on the writing advice.

## Conflict of interest

The authors declare that the research was conducted in the absence of any commercial or financial relationships that could be construed as a potential conflict of interest.

## Publisher’s note

All claims expressed in this article are solely those of the authors and do not necessarily represent those of their affiliated organizations, or those of the publisher, the editors and the reviewers. Any product that may be evaluated in this article, or claim that may be made by its manufacturer, is not guaranteed or endorsed by the publisher.
